# A Vibration-Based Strategy for Health Monitoring of Offshore Pipelines' Girth-Welds

**DOI:** 10.3390/s140917174

**Published:** 2014-09-15

**Authors:** Pejman Razi, Farid Taheri

**Affiliations:** Advanced Composites and Mechanics Laboratory, Department of Civil and Resource Engineering, Dalhousie University, 1360 Barrington Street, Halifax, NS, B3H 4R2, Canada; E-Mail: Pejman.Razi@dal.ca

**Keywords:** vibration-based damage detection, empirical mode decomposition (EMD), submerged pipelines, girth-weld, piezoelectric transducers, energy-based damage index

## Abstract

This study presents numerical simulations and experimental verification of a vibration-based damage detection technique. Health monitoring of a submerged pipe's girth-weld against an advancing notch is attempted. Piezoelectric transducers are bonded on the pipe for sensing or actuation purposes. Vibration of the pipe is excited by two means: (i) an impulsive force; (ii) using one of the piezoelectric transducers as an actuator to propagate chirp waves into the pipe. The methodology adopts the empirical mode decomposition (EMD), which processes vibration data to establish energy-based damage indices. The results obtained from both the numerical and experimental studies confirm the integrity of the approach in identifying the existence, and progression of the advancing notch. The study also discusses and compares the performance of the two vibration excitation means in damage detection.

## 1. Introduction

Offshore pipelines are susceptible to initiation of various types of defects, including corrosion, dents, and cracking/leakage, especially in their mating interfaces (e.g., girth-welds, and bolted joints). Therefore, periodic visual inspections have to be carried out by skilled divers or remote operating vehicles (ROVs) [1]. Such inspections are usually followed by more advanced examinations (e.g., automated ultrasonic technique (AUT) or eddy current method), once any suspected areas are detected.

Health monitoring of a large network of offshore pipelines, even at a preliminary stage (*i.e.*, visual inspection), is usually a cumbersome and costly practice. Vibration-based approaches have therefore been developed and been proven to be relatively successful in detecting damage [1–4]. Therefore, they could potentially reduce the requirement of summoning skilled divers for performing the initial examinations, thus facilitating quicker and more cost-efficient inspections.

To the best knowledge of the authors, there have been very few studies conducted on damage detection of submerged structures; some of the noteworthy ones are briefly mentioned here. Na and Kundu [1] applied the guided wave technique for detection of mechanical defects (e.g., a dent, and removed material) in a scaled submerged pipe. The health monitoring of the pipe was achieved by a transmitter and a receiver, each located on either ends of the pipe. The relatively long range of inspection was noted as the advantage of the method. However, adjustment of the transmitter angle (in a trial and error fashion) was necessitated to achieve a reliable identification of damage in the submerged pipe.

Rizzo *et al.* [5] developed a method for health monitoring of a submerged plate hosting a notch and corrosion. A non-contact pulsed laser unit introduced stress waves into the plate. Two immersion non-contact sensors, made of piezoelectric materials, captured the reflected waves. The plate was scanned along its length by a pulse/sensing unit at discrete locations. The continuous wavelet transform (CWT) was used to extract a damage sensitive parameter from the time modulated frequency domain of the sensors' signals. The method could identify the location of damage by producing relatively large damage indices as the sensing probes passed across the defect regions. They marked the inclination angle along with the relative location of their adopted transducers-damage, and the proximity of the sensors as the influential parameters in the damage detection of the submerged plate.

A research team also conducted a series of extensive numerical and experimental studies to identify free-spanning (a segment of a pipe that is suspended and does not have the seabed or soil support) and corrosion along onshore and offshore pipelines [6–11]. In their study, damage detection was facilitated mainly via two approaches: (i) observation of pipeline's eigenvalues, and (ii) processing of the acceleration time-history data gathered along the pipe as the pipe underwent random or forced vibrations. The team could successfully identify the location of damage. However, there were some inconsistencies in quantifying damage size. Moreover, the free-spanning of pipelines was more reliably detected rather than the corrosion. Furthermore, in some cases, they failed to detect small corrosion; however, free-spanning of pipelines greater than 5% of the pipe's length was confidently identified.

Chen *et al.* [12] developed a strategy for health monitoring of a submerged plate against corrosion. They used piezoelectric transducers to generate and receive lamb waves. The received signals were processed within a probability-based diagnostic imaging approach to identify the damage. The numerical and experimental verification of the method yielded successful results in identifying the damage location.

In the present work, numerical simulations and experimental verification of a vibration-based damage detection methodology (developed in our research group [13]) for health monitoring of a submerged pipe's girth-weld against a propagating notch are presented. The performances of two vibration excitation approaches (*i.e.*, impact and chirp excitation methods) in damage detection are compared. Additionally, the study examines the sensitivity of the method's diagnostic capability to the presence of fluid by which the pipe is pressurized. The paper begins with providing a brief introduction on the vibration-based damage detection strategy adopted in this work. This is followed by the details of the developed numerical model and the experimental setup used in the study. Finally, a discussion on the outcome of the damage detection is presented.

## 2. EMD Energy Damage Index (EMD_EDI)

Huang *et al.* [14] introduced a robust signal processing technique in 1998, which was referred to as the empirical mode decomposition (EMD). In brief, the EMD decomposes a time-domain signal into its oscillatory components referred to as the intrinsic mode functions (IMFs). The decomposition is accomplished through the so-called “sifting process”, which is an empirical and data-driven algorithm [14]. The reader is referred to [15] for a detailed explanation on how the IMFs of a typical signal, that are recorded during a vibration event is extracted. Hilbert transform (HT) is then applied to the IMFs to unveil their time-modulated frequency components. The joint application of the EMD and the HT is called the Hilbert-Huang transform (HHT). The HHT outperforms the wavelet transform (WT), in the sense that it can accommodate both linear and non-linear systems. In addition, it is a data-driven signal processing algorithm, thus its performance would not be affected by pre-defined functions typically used in the fast Fourier transform (FFT) or WT.

Thereafter, the EMD has been used in data interpretation in various disciplines [14]. For instance, it has been shown that the first two IMFs extracted from vibration data of a system would be sensitive to presence of damage [13,16]. On the same basis, Cherghai and Taheri [13] introduced the energy of the IMFs as an efficient and robust damage indicator, defined as follows:

(1) $$E=\int_{0}^{t_{0}}{\left(\text{IMF}1\right)}^{2}dt$$

where *t*_0_ is the signal duration. Subsequently, damage indices can be established as follows:

(2) $$\text{EMD}\_\text{EDI}=\left|\frac{E_{\text{healthy}}-E_{\text{Damaged}}}{E_{\text{healthy}}}\right|\times 100$$

In the above equation, *E_Healthy_*, and *E_Damaged_* are the energy terms calculated from vibration signals of individual sensors gathered at the initial state (considered as the healthy state) and the subsequent (or potentially, the damaged) state of a given structure, respectively. Sensors producing relatively higher damage indices infer presence and the location of damage. Progression of damage can also be quantified by noting the increasing value of damage indices monitored as a function of time. Several experimental case studies accompanied by numerical simulations have been conducted to verify the integrity and effectiveness of the proposed methodology [2–4,15,17]. This method, which was developed in our research group, has been therefore adopted in the current study.

## 3. Finite Element Modeling

### 3.1. Modeling of a Submerged Pipe Equipped with Piezoelectric Transducers

**Figure 1.**
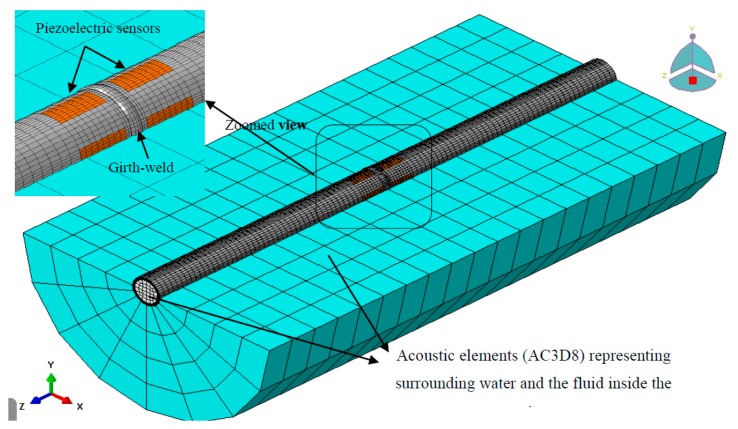
Finite element model of the submerged pipe incorporating piezoelectric sensors.

An aluminum pipe was considered in this case study. The material properties and dimensions of the pipe are listed in Table 1. The pipe was modeled with a total of 33,440 solid elements using ABAQUS's element C3D8R (the continuum three-dimensional eight-node reduced integration element with three translational degrees of freedom per node). Using the same element, a mid-span girth-weld was modeled for the pipe (see Figure 1). A bulge with 10 mm width and 2 mm height was used to model the girth-weld over the pipe circumference, taking into account the local stiffening introduced by the weld material. To account for a clamped-clamped boundary condition, the translational degrees of freedom of the elements at both ends of the pipe were restrained.

To monitor pipe's vibration, eight piezoelectric sensors were bonded to the pipe, 5 mm away from either sides of the girth-weld in the configuration depicted in Figure 1. The sensors' dimensions were measured to be (45 × 20 × 0.15) mm. A total of 64 three-dimensional eight-node piezoelectric elements (C3D8E) were used to model each piezoelectric sensor. The “TIE” constraints were used to connect the sensors and pipe's nodes at their interfaces. The sensors were made of piezoceramics (model: PZT-5H) with the following electro-mechanical properties:

$${\text{S}}_{\text{E}}\left[\frac{{\text{m}}^{2}}{\text{N}}\right]=(10^{-12})\;\left[\begin{array}{cccccc} 16.5 & -4.78 & -8.45 & 0 & 0 & 0\\ -4.78 & 16.5 & -8.45 & 0 & 0 & 0\\ -8.45 & -8.45 & 20.7 & 0 & 0 & 0\\ 0 & 0 & 0 & 43.5 & 0 & 0\\ 0 & 0 & 0 & 0 & 43.5 & 0\\ 0 & 0 & 0 & 0 & 0 & 42.6 \end{array}\right]$$

$$\text{d}\left[\frac{\text{C}}{\text{N}}\right]=(10^{-12})\;\left[\begin{array}{cccccc} 0 & 0 & 0 & 0 & 741 & 0\\ 0 & 0 & 0 & 741 & 0 & 0\\ -274 & -274 & 593 & 0 & 0 & 0 \end{array}\right]$$

$${ɛ}^{\text{S}}[\text{F}/\text{m}]=(10^{-9})\;\left[\begin{array}{ccc} 27.7 & 0 & 0\\ 0 & 27.7 & 0\\ 0 & 0 & 30.1 \end{array}\right]$$

where *S_E_* is the compliance matrix, *d* is the piezoelectric coupling matrix, and ε*^S^* is the permittivity of the piezoceramic. The density of the piezoceramic was taken as 7500 kg/m^3^. Individual piezoelectric transducers could serve as a sensor and an actuator. They could turn into an actuator once a surface charge is defined for one of their surfaces.

ABAQUS's acoustic element, AC3D8 (an eight-node three-dimensional acoustic element) was used to model the surrounding water and the internal fluid in the computational domain; 1280 and 640 elements constructed the medium and the internal fluid, respectively. Density and bulk modulus values of water were taken as 997 kg/m^3^, and 2.13 GPa, respectively, and 1.2 kg/m^3^ and 101,000 Pa for air. The “TIE” constraints were also used to form the interfaces of the pipe and external and internal fluids. The constraints correlated the pipe's surface displacement degrees of freedom to the neighboring fluid elements' pressure degrees of freedom [18].

The question was raised whether one could model the pipe within an effective water depth (EWD), as opposed modeling a large volume of water, hence reducing the computational cost without sacrificing the accuracy. To establish the appropriate EWD, one can establish a boundary such that the reflected waves from that boundary would not affect the pipe's vibration. In other words, the reflection from the boundary would become negligible once the boundary is located adequately far from a vibrating structure [18].

**Figure 2.**
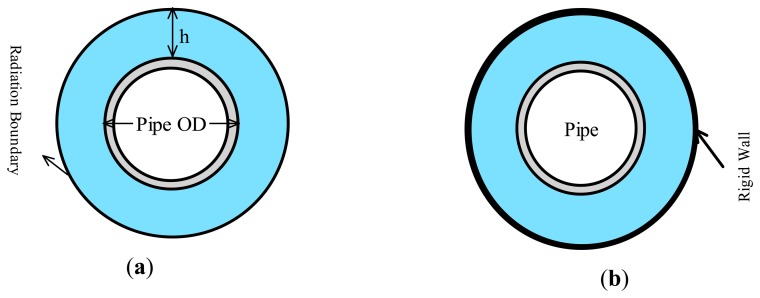
A submerged pipe with: (**a**) radiation and (**b**) rigid wall boundaries.

Two different boundary conditions were considered as the exterior boundaries of water in order to establish the EWD; they were (i) radiation and (ii) rigid-wall (see Figure 2). The radiation boundary would facilitate transmission of the acoustic waves across the boundaries with little reflection of energy back into the acoustic domain. On the other hand, the acoustic waves would reflect back into the acoustic domain after they hit the rigid-wall boundary [18]. Subsequently, the variation of the first-three natural frequencies of the submerged pipe against the step-wise levels of submergence, *h/OD*, was observed. *h* is the height of water above the pipe, and *OD* is the pipe's outer diameter.

**Figure 3.**
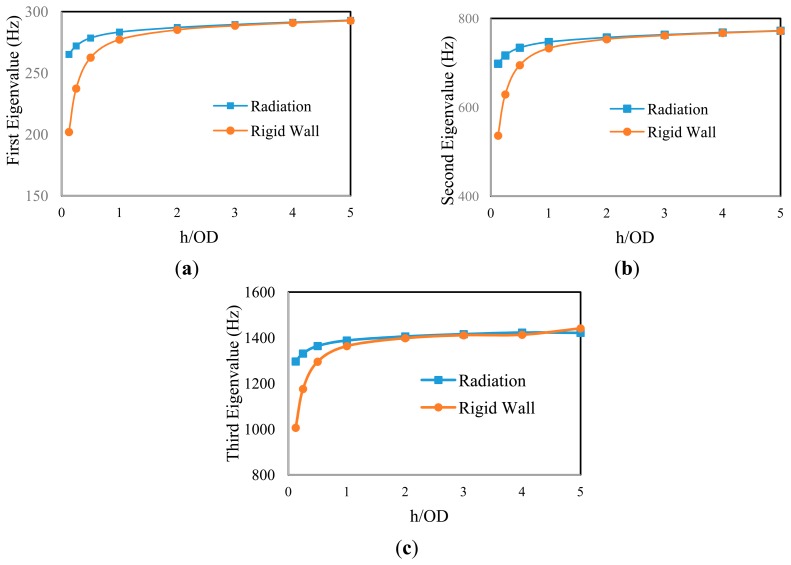
Evolution of the pipe's eigenvalues: (**a**) first eigenvalue, (**b**) second eigenvalue, (**c**) third eigenvalue.

The results shown in Figure 3 indicate that one could assume an EWD equal to four times the outer diameter of the pipe, beyond which, the boundary-type would not affect the natural frequencies of the pipe; therefore, *EWD = h/OD =* 4 is considered in the analysis, hereafter.

In addition, a mesh convergence study was conducted to ensure the accurate representation of the submerged pipe model. The mesh density was increased to a level such that the variation in the first-three eigenvalues remained below 1%.

### 3.2. Transient Analysis

**Figure 4.**
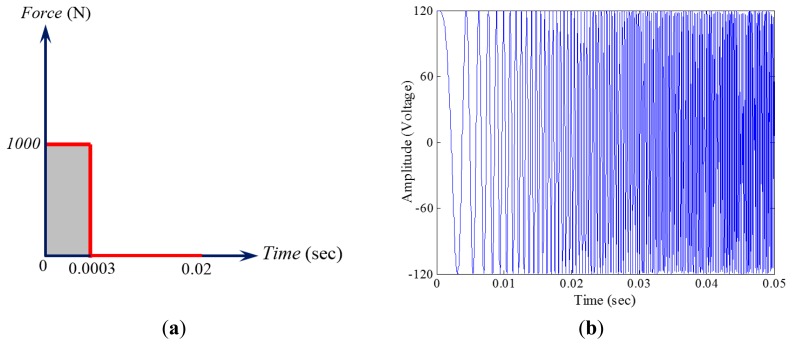
Schematic representations of (**a**) the impact and (**b**) the chirp excitations.

Health monitoring of the submerged pipe was accomplished by conducting a series of transient dynamic analysis solved by the implicit solution algorithm of the ABAQUS. Two excitation approaches were adopted in the damage detection trials. First, an impulse force, similar to the force that was produced by a pneumatic hammer that was used in the experiment, was prescribed to the pipe by defining a force with magnitude of 1000 N; this force was assumed to be acting over a very short period of time (0.0003 s). The other alternative excitation method was achieved by using one of the piezoelectric transducers as an actuator. In this way, a chirp signal was propagated into the pipe by the actuator. The adopted chirp, defined as a surface charge in the piezoelectric actuator, contained a frequency range of 10–5000 Hz, varied linearly over 0.05 s. The schematic representations of the actuation units are elucidated in Figure 4.

The transient analysis was performed with an increment of 0.00002 s. In the other words, the pipe's vibration was sampled at 50 kHz (1/0.00002 s), so that it could accommodate the highest excitation frequency (*i.e.*, 5 kHz) through the chirp. The pipe's vibration was registered via the piezoelectric sensors' output voltage.

The analysis run-time was set to 0.02 s and 0.05 s for the tests conducted by the impact and the chirp excitation methods, respectively. It should be mentioned that a chirp signal with the same frequency content, but varying over 0.5 s (similar to the experimental study), was initially considered. However, a time-sensitivity analysis was performed and revealed the insensitivity of the damage detection's outcome to the selected time modulation of the chirp signal. Therefore, the aforementioned chirp was applied over 0.05 s in order to reduce the computational cost without sacrificing the accuracy.

## 4. Experimental Framework

**Figure 5.**
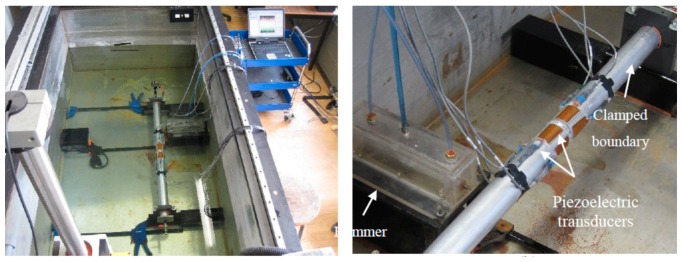
Schematic of the experimental setup for conducting the damage detection trials.

An aluminum pipe with the same material properties and dimensions listed in Table 1 was considered for the experimental segment of the work. The tests were conducted in a 2.45 × 1.12 × 0.78 m^3^ laboratory tank. The pipe was clamped at both ends as shown in Figure 5. A pressure gauge, mounted on the pipe's cap, was used to monitor the internal fluid pressure.

Eight flexible piezoelectric actuator/sensors (model: pa16n, Mide Technology Corporation, Medford, MA, USA) were bonded to the pipe in either sides of the girth-weld as shown in Figure 5b. The transducers were enclosed by a special waterproof coating; however, the electrical connections were sealed manually by applying a generous amount of silicon, and then each individual connection was further secured by heat shrink sleeves.

**Figure 6.**
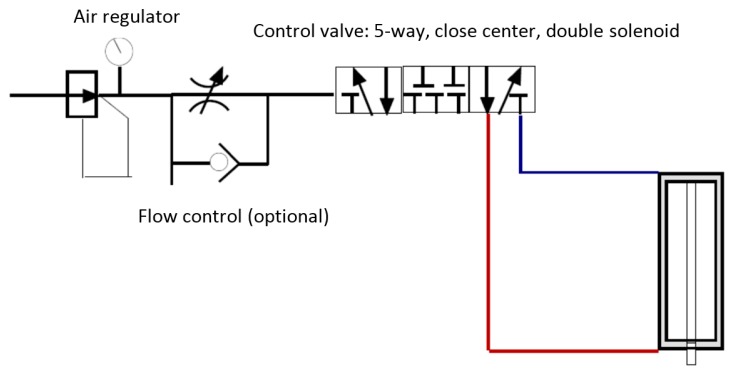
Schematic design of the pneumatic hammer.

As stated earlier, two excitation methods were considered for conducting the damage detection trials:

(1) Impact
  A waterproofed pneumatic hammer was designed and fabricated in-house to generate the required impulsive forces. Figure 6 shows the symbolic design of the hammer. An air regulator assured the flow of air with a constant pressure, thus ensuring the consistency of the impulse load. A flow control tuned the intensity of the impulse load. Back and forth strikes of the piston was controlled by a solenoid valve through an electrical switch. The cylinder and piston assembly was waterproofed by incorporating a Plexiglas box. An O-ring sealed the box/piston gap. A close-up of the fabricated hammer is shown in Figure 5b.
(2) Chirp waves
  The second actuation means was accomplished by using one of the piezoelectric transducers as an actuator, thus propagating chirp waves in the pipe. A signal generator (model 33210A, available from Agilent Technologies, Santa Clara, CA, USA) was used to generate the chirp signals. The signals were then amplified via a power amplifier (model: 790 series available from PCB Piezotronics, Inc., Depew, New York, NY, USA), before being injected to the piezoelectric actuator. The chirp signals contained frequency range of 10–5000 Hz, varied linearly over 0.5 s. The piezoelectric-based excitation method provides the following main advantages over the impulsive excitation, as typically generated by an impact hammer:
  - It effectively manages the consistency of excitation, thus minimizing the discrepancy in repeated trials' measurements; the force generated by an impulse hammer is vulnerable to severe inconsistencies due to several factors, including small deviations in the impact location and the magnitude of generated force.
  - It allows exploitation of a wide range of frequencies, and selection of various types of excitation signals (*i.e.*, chirp, burst, and random).

The vibration signals gathered through the sensors were digitized by a data acquisition system (NI-9215 in a compact chassis manufactured by National Instrument Inc., Austin, TX, USA). The sampling rate was set to 50 kHz.

## 5. Model Verification

An experimental study was conducted in our laboratory tank to verify the integrity of the developed numerical model before beginning the damage detection process. For that, the experimentally measured eigenvalues of a submerged pipe were compared against those obtained from the simulation. The aluminum pipe was hanged in the tank by soft elastic ropes emulating a free-free boundary condition. One of the piezoelectric transducers was used to propagate the chirp signal along the pipe. The pipe's vibration was recorded by one of the piezoelectric sensors, whose output was a voltage-signal. The experiment was simulated by the numerical model described earlier. The eigenvalues of the submerged pipe were determined by applying the FFT to the pipe's forced vibration signals obtained from the experimental study and the model. The first-three eigenvalues of the submerged pipe, obtained via the two approaches, were compared as tabulated in Table 2. The reasonable agreement between the experimental and numerical results confirms the integrity of the developed model.

## 6. Damage Scenarios

**Figure 7.**
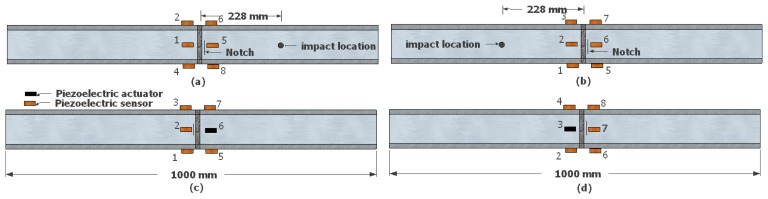
Schematic view of damage scenarios. The pipe was pressurized by air ((**a**) and (**c**)) and water ((**b**) and (**d**)).

Health monitoring of the pipe's girth-weld was attempted by utilizing the outlined damage detection algorithm. A notch of 1 mm depth (*i.e.*, 19% of the wall thickness) was introduced adjacent to the girth-weld and was then propagated to a depth of 4 mm (*i.e.*, 77% of the wall thickness) in 1 mm increments. In the experiments, the notches were created by a jeweler saw. Two different conditions were considered for the pipe's internal loading conditions: (i) the pipe pressurized with air (P = 1 MPa) and (ii) with water (P = 5 MPa). Figure 7 depicts the notch locations and the arrangements of sensors/actuators in the four damage scenarios considered in this study. The experimental framework also incorporated the above-mentioned excitation methods for comparative purposes.

**Figure 8.**
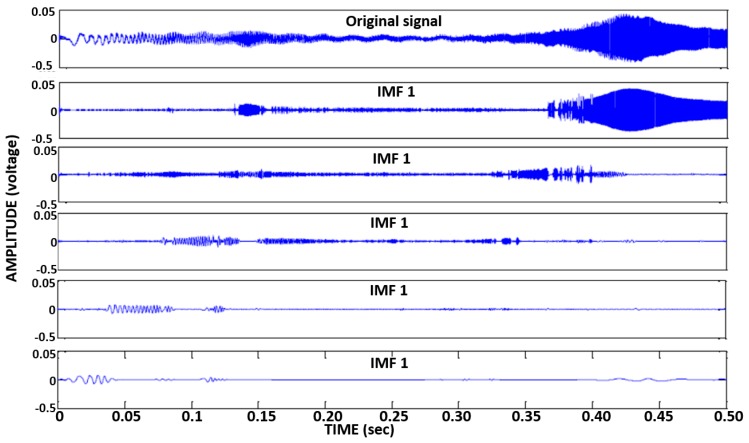
A typical sensor's vibration response to the chirp (original signal) and its first five IMFs obtained through EMD.

The numerical simulations included execution of a transient analysis to capture the dynamic response of the pipe at its initial state (*i.e.*, the healthy state), as well as for each incremental damaged state. The vibration signals at each run were recorded through the sensors. An in-house MATLAB-code was developed to include the adopted damage detection algorithm (EMD_EDI). The code performed the following tasks on the vibration data:

(i) Filtered the signals (the code applied low-pass filters of [0–1000] Hz and [0–5000] Hz to the signals obtained due to the application of the impulse load and chirp excitation, respectively.)
(ii) Extracted the IMFs of vibration signals through EMD (see Figure 8)
(iii) Calculated the first IMF's energy (using Equation (1))
(iv) Established the damage indices (using Equation (2))

## 7. Results and Discussions

In this section, the results of the damage detection trials conducted by the two excitation technique are presented and the outcomes are discusses.

### 7.1. Damage Detection Results-Impact Test

**Figure 9.**
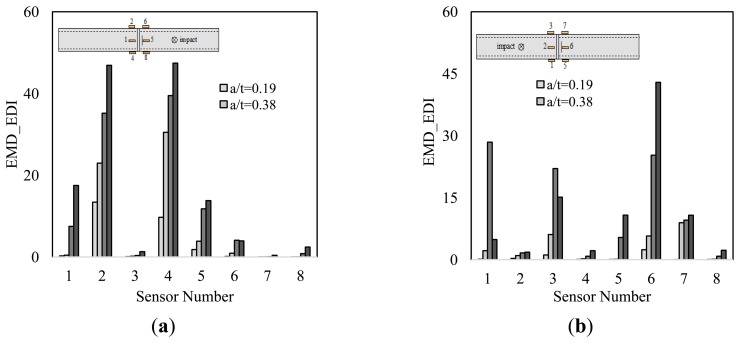

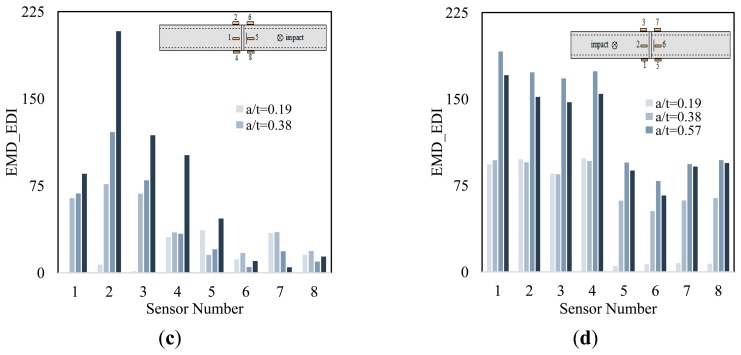
Damage indices obtained from the impact test: numerical ((**a**) and (**b**)) and experimental study ((**c**) and (**d**)). Note that a/t is the ratio of notch's depth to pipe's wall thickness.

The charts shown in Figure 9 illustrate the results of the damage detection conducted by the impact test. The results obtained from both the numerical and experimental studies indicate that at least half of the sensors could effectively detect the existence of the damage (*i.e.*, notch), and trace its advancement. Half of the sensors yielded higher damage indices as the notch depth increased. The only exception was in the case shown in Figure 9d. In that case, while the existence of the notch was identified with notably large indices, its advancement, however, was underestimated at its final increment. The rest of the sensors could also discern the existence of the notch; however, they occasionally failed to predict its advancement.

It can be inferred from the results that the relative location of the sensor and actuators with respect to the notch is responsible for the variability seen in the results. Those sensors receiving their vibration waves passing through the notch outperformed the rest of the sensors in terms of damage detection accuracy. This statement can be further explained by referring to Figure 9b, which presents the results of the damage detection for the scenario considered in Figure 7b. The figure shows that signals of sensors 5 to 8 were able to trace the notch advancement, while those of sensors 1 and 3 failed to do so. In Figure 9a, EMD_EDIs of sensors 1–4 signals, which conform to the stated condition, produced noticeably higher damage indices, as well as providing a clearer indication of damage advancement.

Based on the analysis of the results, one can postulate that the diagnostic capabilities of half of the sensors were affected by the reflection of the waves from the notch. Conducting a systematic parametric study on pipes with larger diameters could shed more light on the observed behavior and validity of the noted postulation.

### 7.2. Damage Detection Results-Chirp Test

The results of the damage detection tried by the chirp method qualitatively confirm the findings of our previous trial, as noted above (*i.e.*, when the pipe was excited by an impact). Comparatively, the advancement of the notch could be traced more accurately when the chirp method is used to excite the pipes.

### 7.3. Comparison of the Application of Impact and Chirp

**Figure 10.**
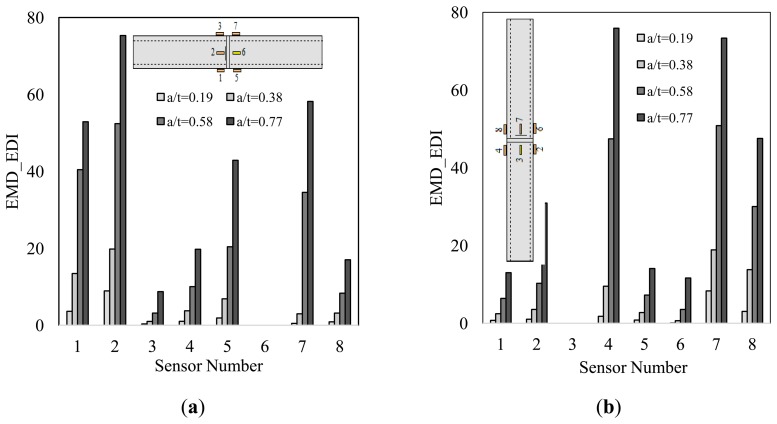

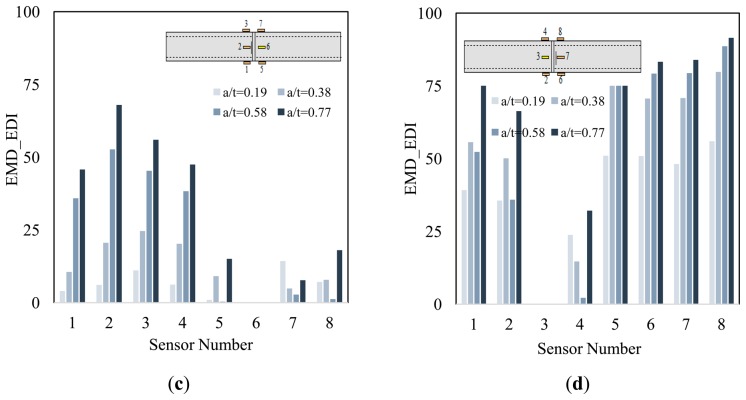
Damage indices obtained from the chirp test: numerical ((**a**) and (**b**)) and experimental study ((**c**) and (**d**)). Note that a/t is the ratio of notch's depth to pipe's wall thickness.

The diagnostic capability of the method in damage localization was also investigated under the both excitations approaches. The numerical results illustrated in Figure 9a,b, and Figure 10a and b suggest that the closest sensor to damage produced the highest damage index; hence, it can be concluded that an accurate damage localization could also be produced by the presented methodology. In consideration of the results, the only exception applies to the first damage scenario (see Figure 9a). In that situation, the closest sensor to the damage (*i.e.*, sensor 5) did not produce the largest EMD_EDI. This is because, it was located in-between the source of excitation and the notch, thus a low diagnostic capability was already anticipated based on the observations explained in Section 7.1.

In comparison to the numerical results, the EDIs obtained by processing of the experimental data produced somewhat controversial results in terms of damage localization. The results presented in Figure 10c show that signals collected through sensor 2 (the closest sensor to the damage) yielded the largest EMD_EDI, hence identifying the exact location of the notch on pipe's circumference. For the remaining damage scenarios, the location of damage could not be discerned successfully. It is believed that the inevitable uncertainties involved in the experiments (e.g., the variable pipe/sensor's bond-strength, and the minute inequalities in sensors/girth-weld distances, which occurred unintentionally during the process of bonding the sensors to the pipe) could have affected the accuracy of damage localization.

Taking the above observations into account, it could be suggested that one requires a minimum of two piezoelectric sensors for accurate health monitoring of such damage conditions (such that one sensor is bonded on each side of the girth-weld, opposite to one another with respect to the axial direction). A more effective health monitoring, however, could be achieved by conducting two separate trials, using either of the excitation techniques. In the case of vibration excitation by an impulse force, the two tests would consist of impacting the pipe at both sides of the girth-weld, and recording the associated vibration of each impact. In the case of chirping, the sensors can alternate their tasks in the form of a sensor and actuator, respectively. In this way, upon completion of the two tests, the existence and severity of damage could be more effectively discerned by noting the largest EMD_EDI obtained from the two trials. The conclusion holds also true for the numerical model, so long as the chirp excitation method is used as the means of excitation; otherwise, at least four sensors (two placed on each side of the girth-weld) are required for a successful damage detection, since it was observed that some of the sensors could not sense the existence of the damage (see Figure 9a,b).

In all, both the numerical and experimental studies provided satisfactory results with respect to identification of an advancing notch, regardless of the fluid-type the pipe carried (*i.e.*, compressible (air) or incompressible (water)). It can therefore be concluded that the variable damping introduced by the presence of fluid inside the pipe would not weaken the method's diagnostic capability.

Moreover, the chirp excitation method generally outperformed the commonly-used impulsive force excitation technique, in terms of both damage localization and prediction of its advancement. In addition, the results obtained through the sensors in the experimental studies managed to detect the onset and advancement of damage with larger EMD_EDIs compared to the EMD_EDIs obtained through the numerical simulations. As for the noted discrepancies between the results of the experimental and numerical investigations utilizing the impact hammer, the reason could be due to the difference in the actual time-domain history of the applied impulse forces. It should be noted that since there was no direct means to measure the magnitude of the force during the tests, therefore, an approximate loading amplitude (based on our previously conducted trials and experience with a modal hammer) was selected for replicating the impulse force in the numerical study. Furthermore, due to unavoidable circumstance, the pneumatic hammer produced double-impact at each loading application. As a result, the time resolution of the repeated force could not be retrieved accurately within each test. Therefore, it was idealized as a single impulse force in the numerical simulations.

### 8. Remarks on the Environmental/Operational Conditions

In the experimental investigation, the repeatability of the measurements was established by comparing the energy terms resulting from the applied excitation from one trial to the next for total of ten trials. The consistency of the impact force was determined to be 92% and 98% for the pneumatic hammer and piezoelectric actuator, respectively. The deviations in the measurements are believed to be due the inevitable noise associated with the instruments and laboratory noise, as well as due to minor inconsistencies produced during the hammer impacts.

**Figure 11.**
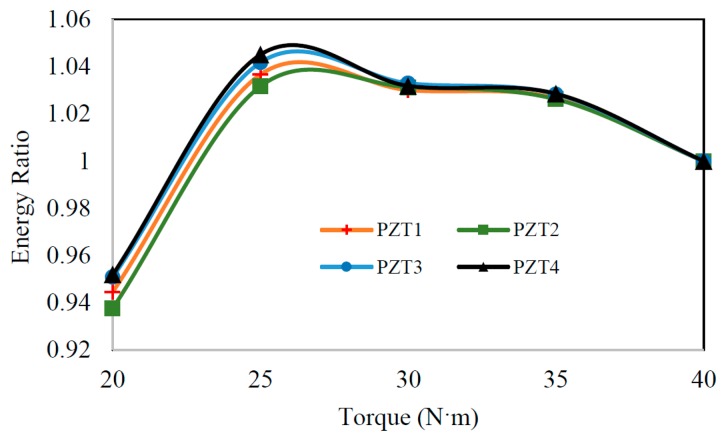
The normalized energy as a function of the clamp torque level.

It was also of interest to examine the simultaneous effects of the noise and disturbance associated to the pipe's constraints (which could occur in real applications) on the repeatability of the measurements. For that purpose, the clamping torque of the bolts securing the collar on one of the pipe's ends tested in air (see Figure 5) was reduced from (40 to 20) N·m in increments of 5 N·m. The precise measurement of the clamping forces on the bolted assembly was facilitated using a digital torque meter. A chirp-type wave was propagated via piezoelectric sensor 5 at each individual torque level, and the vibration response was recorded by the first four sensors (sensor 1–4). The energy of each sensor's vibration signal at the specified clamping forces was calculated according to Equation (1), and was subsequently normalized with respect to the energy corresponding to the maximum torque level (*i.e.*, 40 N·m). Figure 11 reports the average of ten measurements at the specified torque levels. As can be seen, the maximum variation in the calculated energies is only 6%. One can therefore make an implicit conclusion that EMD_EDIs with values above 6% would indicate the existence of a damage within a given trial. EMD_EDIs below this threshold could not be attributed to the presence of damage with confidence, since they might be produced as a result of a disturbance in the boundary conditions.

Moreover, owing to the damping of the propagated waves, it is anticipated that the observed variations would be smaller for cases where the supports are located farther from the inspection zone.

Operational variability such as abrupt changes in the internal pressure of the pipe could also affect the integrity of a vibration-based damage detection trial. The additional stiffening generated by the internal pressure can variably modify the overall stiffness of the pipe, thereby its vibration response. As a result, erroneous energies, causing false alarms, could be developed. To investigate the criticality of this issue, the sensitivity of the proposed energy index to such operational variability was examined. For that, different levels of pressures (*i.e.*, 0–5 MPa) were assigned to the internal surface of the healthy pipe. At each pressure level then, the pipe was excited by an impulse load and a chirp wave according to the configuration elucidated in Figure 7b and d, respectfully, and pipe's vibration was recorded by the sensors. The energy of the vibration signal obtained through sensor 5 was calculated according to Equation (1). The energy terms were then normalized with respect to the energy of the sensor 5's vibration signal when the pipe experienced the highest pressure (*i.e.*, 5 MPa).

**Figure 12.**
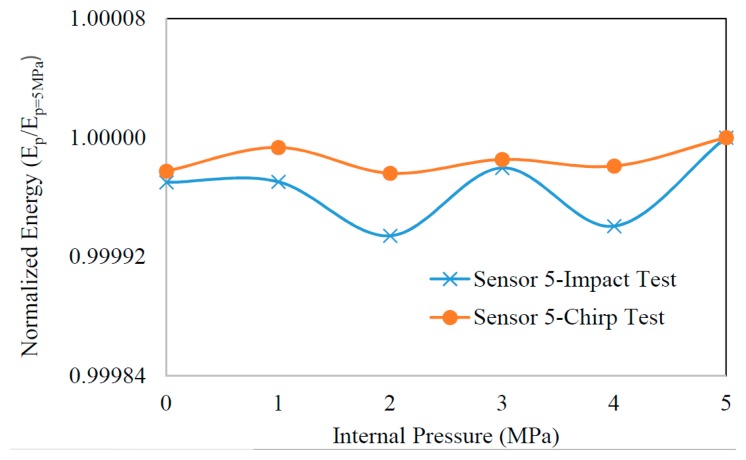
The normalized energy of the vibration signal as a function of internal pressure.

Figure 12 shows the fluctuations of the energy as a function of the applied internal pressure. It can be seen that the rate of variations in the energy was limited to below 0.1%. Therefore, the study indicated that the pipe vibration remained almost unaffected under the imposed operational variability. As such, the proposed vibration-based damage detection algorithm can be reliably applied to the pipe in the presence of such variations.

## 9. Conclusions

Numerical simulations and experimental verifications of a vibration-based damage detection strategy for health monitoring of submerged pipelines' girth-welds were presented. Piezoelectric transducers were used in the capacity of actuators and sensors to excite and record pipe's vibration, respectively. The damage detection methodology incorporated the empirical mode decomposition (EMD) to process the recorded vibration signals and establish the energy-based damage indices (EMD_EDIs). The integrity and efficiency of the technique were evaluated by detection of an advancing notch that was formed in the girth-weld of two mating aluminum pipes. The results of the numerical study were compared against those obtained from the experimental investigations. Reasonable agreement was obtained between the damage detection indices produced by the numerical and experimental case studies. Examination of the EMD_EDIs produced through sensors' data revealed encouraging evidence with respect to detection of the presence and advancement of damage, regardless of the type of fluid used to pressurize the pipe.

The investigation also examined the performance of two vibration excitation approaches, namely: the impact method and chirp excitation method. The results demonstrated the effectiveness of the chirp excitation method over the impact method for damage detection of the submerged pipes. It was also revealed that method could sustain its integrity and reliability in the presence of some common environmental and operational variability (e.g., noise, disturbance in boundary conditions, and abrupt changes in the internal pressure). Finally, it was concluded that health monitoring of the submerged pipe's girth-weld could be efficiently accomplished with a minimum of two transducers, one acting as a sensor and the other as an actuator, bonded on each side of the girth-weld.

## Figures and Tables

**Table 1.** Dimensions and mechanical properties of the aluminum pipe.

**Mechanical Properties**	
Elastic modulus (GPa)	68.9
Density (kg/m^3^)	2700
Poisson's ratio	0.33

Dimensions (m)	

Length	1
Outer diameter	0.06
Wall thickness	0.0052

**Table 2.** Eigenvalues of the submerged pipe.

**Eigenvalues (Hz)**	**Experiment**	**Numerical Model**	**Difference (%)**
f_1_	200.0	198.5	0.7
f_2_	542.8	533.0	1.8
f_3_	1030.6	1002.5	2.7
